# Supplementary material for: Changes in the free-energy landscape of p38α MAP kinase through its canonical activation and binding events as studied by enhanced molecular dynamics simulations
Source: eLife. 2017 Apr 26;6:e22175. doi: 10.7554/eLife.22175 (PMC5406204; doi:10.7554/eLife.22175)
Supplement: Supplementary file 1. — Residue pairs with interaction occupancy >75% in the most populated clusters of the selected minima are bolded, while the occupancy for the rest of the pairs is in the range of 60–75%. DOI: http://dx.doi.org/10.7554/eLife.22175.020 [file elife-22175-supp1.docx]

| System | Minimum | Residue pairs |
| --- | --- | --- |
| p38α | 2-4 kcal mol^-1^ | **D168-R173**, **R173-E71** |
|  | 4-6 kcal mol^-1^ | **D168-R173**, **D176-R149**, **D177-R70**, **E178-R173**, **E178-R67** |
| p38α-pTpY | 4-6 kcal mol^-1^ (CV_1_, CV_2_ = 0.15, 0.75) | **R173-E71**, **pT180-R189** |
|  | 16-18 kcal mol^-1^ | **D177-R149**, **pT180-R173**, **pT180-R67**, **pT180-R70**, **D177-R173**, **E178-K66**, **E178-R70** |
| p38α-pTpY (ATP) | 2-4 kcal mol^-1^ | pT180-R173, pT180-R67, pT180-R70, **pY182-R149** |
|  | 4-6 kcal mol^-1^ | D176-R189, **D177-R149**, **E178-R173** |
